# New Books

**Published:** 2011-06

**Aerosol Measurement: Principles, Techniques, and Applications, 3rd ed.**

Pramod Kulkarni, Paul A. Baron, Klaus Willeke, eds.

Hoboken, NJ:John Wiley & Sons, 2011. 900 pp. ISBN: 978-0-470-38741-2, $195

**Analysis of Chemical Warfare Degradation Products**

Karolin K. Kroening, Renee N. Easter, Douglas D. Richardson, Stuart A. Willison, Joseph A. Caruso

Hoboken, NJ:John Wiley & Sons, 2011. 160 pp. ISBN: 978-0-470-74587-8, $95

**Applications of Toxicogenomics in Safety Evaluation and Risk Assessment**

Darrell R. Boverhof, B. Bhaskar Gollapudi, eds.

Hoboken, NJ: John Wiley & Sons, 2011. 396 pp. ISBN: 978-0-470-44982-0, $115

**Bringing Ecologists and Economists Together**

T. Söderqvist, A. Sundbaum, C. Folke, K.-G. Mäler, eds.

New York:Springer, 2011. 281 pp. ISBN: 978-90-481-9475-9, $129

**Chemical Biomarkers in Aquatic Ecosystems**

Thomas S. Bianchi, Elizabeth A. Canuel

Princeton, NJ:Princeton University Press, 2011. 392 pp. ISBN: 978-1-4008-3910-0, $95

**Climate and the Oceans**

Geoffrey K. Vallis

Princeton, NJ:Princeton University Press, 2011. 232 pp. ISBN: 978-0-691-14467-2, $80

**Climate Change Justice**

Eric A. Posner, David Weisbach

Princeton, NJ:Princeton University Press, 2010. 240 pp. ISBN: 978-1-4008-3440-2, $27.95

**Emergency Response Management of Offshore Oil Spills: Guidelines for Emergency Responders**

Nicholas P. Cheremisinoff, Anton Davletshin

Hoboken, NJ:John Wiley & Sons, 2010. 529 pp. ISBN: 978-0-470-92712-0, $175

**Encyclopedia of Bioterrorism Defense, 2nd ed.**

Rebecca Katz, Raymond A. Zilinskas, eds.

Hoboken, NJ:John Wiley & Sons, 2011. 688 pp. ISBN: 978-0-470-50893-0, $449.95

**Energy for a Sustainable World: From the Oil Age to a Sun-Powered Future**

Vincenzo Balzani, Nicola Armaroli

Hoboken, NJ:John Wiley & Sons, 2011. 390 pp. ISBN: 978-3-527-32540-5, $45

**Everyday Environmentalism: Law, Nature, and Individual Behavior**

Jason J. Czarnezki

Washington, DC:Island Press, 2011. 150 pp. ISBN: 978-1-58576-152-4, $29.95

**Indoor Air Pollution**

Robert Maynard, Paul Harrison, Isabella Myers, eds.

Hackensack, NJ:World Scientific, 2011. 400 pp. ISBN: 978-1-84816-493-2, $130

**The Economics of Enough: How to Run the Economy as If the Future Matters**

Diane Coyle

Princeton, NJ:Princeton University Press, 2011. 336 pp. ISBN: 978-0-691-14518-1, $24.95

**The Rising Sea**

Orrin Pilkey, Rob Young

Washington, DC:Island Press, 2011. 224 pp. ISBN: 978-1-61091-004-0, $22.95

